# Voting with your wallet? Municipal budget policy and election results

**DOI:** 10.1371/journal.pone.0221619

**Published:** 2019-08-29

**Authors:** Stijn Baert, Herman Matthijs, Ilse Verdievel

**Affiliations:** 1 Ghent University, Ghent, Belgium; 2 Research Foundation–Flanders, Brussels, Belgium; 3 University of Antwerp, Antwerp, Belgium; 4 Université Catholique de Louvain, Louvain-la-neuve, Belgium; 5 IZA, Bonn, Germany; 6 GLO, Maastricht, Netherlands; 7 IMISCOE, Rotterdam, Netherlands; 8 Vrije Universiteit Brussel, Brussels, Belgium; Universidad Loyola Andalucia Cordoba, SPAIN

## Abstract

In this research letter, we examine the impact of municipal budget policy on the percentage of votes for the incumbent majority parties in subsequent elections. We contribute to the academic literature by examining the combined influence of taxes, expenditures and debt. Based on data for Flanders (Belgium) between 1994 and 2012, we find no significant association between these budget variables and the actual election results.

## 1. Introduction

In recent decades, various contributions to the field of political economics have focused on the association between budgetary policies and election results at the municipal level. As indicated in Table 1, the results in this literature are variable. For example, studies by Bosch and Sollé-Olé [1] and by Veiga and Veiga [2] report that, in Portugal and Spain, incumbent majorities are punished for increased taxes and rewarded for additional investment expenditures. In contrast, Van Malderen and Gérard [3] find no association between the amount of or developments in income taxes or property taxes and election results at the municipal level in Wallonia (Belgium). At the same time, recent studies have focused on the effect of budget policies in neighbouring municipalities (‘yardstick voting’). The *a priori* expectation of this type of research is that voters will punish their own municipal governments if the policies in the neighbouring municipalities are more favourable by comparison. This expectation is confirmed for Spain and France in studies by Bosch and Sollé-Olé [1] and by Dubois and Paty [4]. These authors report evidence that higher taxes within a voter’s own municipality have a negative impact on the election results of the incumbent majority, as well as evidence that higher taxes in the neighbouring municipalities have a positive impact.

**Table 1:** Literature overview.

Study	Data	Method of Analysis	Budgetary policy elements examined (effect on the percentage of votes for the majority parties in subsequent elections)
Bosch & Sollé-Olé [1]	Spain, 1991–2003	2SLS	Developments in property taxes (-), developments in property taxes in neighbouring municipalities (+)
Brender [14]	Israel, 1983–1998	Logistic regression	Debt (0)
Drazen & Eslava [15]	Colombia, 1987–2002	FE	Investments (+), budget deficits (-)
Dubois & Paty [4]	France, 1989–2001	2SLS	Property taxes (-), property taxes in similar neighbouring municipalities (+)
Van Malderen & Gérard [3]	Wallonia (Belgium), 2006–2012	2SLS	(Developments in) income taxes (0), (developments in) property taxes (0), (developments in) income taxes in neighbouring municipalities (0), (developments in) property taxes in neighbouring municipalities (0)
Veiga & Veiga [2]	Portugal, 1979–2001	FE	Investment expenditures (in election years) (+)
Vermeir & Heyndels [5]	Flanders, 1982–2000	FE/2SLS	Income taxes (0/-), property taxes (0/-), expenditures (0), income taxes in neighbouring municipalities (0/+), property taxes in neighbouring municipalities (0), expenditures in neighbouring municipalities (0)

Notes. FE stands for ‘fixed effects’ (linear regression with fixed effects at the level of the municipality or, in Drazen and Eslava [15], at the party-state level), and 2SLS stands for ‘two-stage least squares’ (instrumental variable estimates). In the fourth column, ‘-‘ (‘0’) ((‘+’)) represents a negative (neutral) ((positive)) association with the percentage of votes for the majority parties. A combination of these symbols (e.g. ‘0/-‘) indicates different results for different methods of analysis.

All of the aforementioned studies focus on particular facets of municipal budgetary policy, while ignoring other facets. Policies concerning municipal income and expenditures (and the resulting debt level) are nevertheless closely related to each other. For example, increased taxes could be expected to be punished less if they lead to popular investments or reductions in the debt level. It therefore seems necessary to consider both of these factors together when examining the association between budgetary policy and election results at the municipal level. In econometric terms, this can cause an ‘omitted variable bias’ (i.e. distortion due to not controlling for variables that are associated with both the independent and the dependent variable).

In this study, we contribute to the literature by examining the effect of the development of municipal taxes, expenditures and debt throughout a legislative period on the election results for the majority parties. To this end, we present estimates that control for fixed effects at the municipal level, as well as instrumental variable estimates.

## 2. Methods

### 2.1 Model

In our econometric analyses, we build on the model developed by Vermeir and Heyndels [5]. More specifically, we estimate the following choice function:

$$V_{it}^{\left[t-1,t\right]}=\alpha V_{it-1}^{\left[t-1,t\right]}+\beta F_{it}+\gamma \sum_{j=1}^{n}w_{ij}\;F_{jt}+\delta X_{it}+{\mu Y}_{t}+\pi P_{it}+\varepsilon_{it}.$$ (1)

The dependent variable in this expression, $V_{it}^{\left[t-1,t\right]}$, stands for the percentage of votes that an incumbent majority (all parties together) in municipality *i* receives in year *t* (i.e. the elections following their policy term [*t*−1,*t*]). $V_{it-1}^{\left[t-1,t\right]}$ is the percentage of votes for the same parties in the previous municipal elections. *F*_*it*_ is a vector of variables that capture the budgetary policy in the municipality at the end of the policy term and $\sum_{j=1}^{n}w_{ij}\;F_{jt}$ represents the mean of the same variables in the adjacent municipalities (i.e. *w*_*ij*_ equals 1 divided by the number of adjacent municipalities in case *i* and *j* are adjacent and 0 otherwise). *X*_*it*_, *Y*_*t*_ and *P*_*it*_ are vectors of control variables: municipal characteristics, year dummies and party-year dummies–capturing the six main national parties in majorities: Open VLD, N-VA, CD&V, Sp.a, GROEN and Open VLD–respectively. *α*, *β*, *γ*, *δ*, *μ* and *π* are the respective (vectors of) coefficients to be estimated. In an initial approach, expression (1) is estimated by means of a linear regression (ordinary least squares; OLS).

We adopt two main strategies to control for further determinants of the dependent variable that cannot be observed in the research data. In the first strategy, we perform linear regressions in which we control for fixed effects (FE) at the level of the municipality. With this approach, we filter out all time-constant drivers of election results. In the second strategy, we perform instrumental variable (IV) regressions. In the first step of the latter strategy, *F*_*it*_ and $\sum_{j=1}^{n}w_{ij}\;F_{jt}$ are predicted according to a number of instruments: variables that are assumed to have a direct effect on the budget variables but not on $V_{it}^{\left[t-1,t\right]}$ (after controlling for the other variables included).

### 2.2 Data

The choice function in (1) is estimated for the municipal elections in Flanders in October 2000, 2006 and 2012 –the budgetary policy variables mentioned below were not registered uniformly before. Descriptions of the most important variables included are presented in Table 2. These variables were collected and merged from the following sources: the research section of the Flemish government, the election database of the Belgian Federal Public Service Interior and Statistics Belgium (coordinated by the Federal Public Service Economy).

**Table 2:** Data description.

Variable	Description	Source	Mean	Standard deviation
Percentage of votes for majority parties	Total percentage of votes for the incumbent majority in the elections of 2000, 2006 and 2012 (relative to the total number of votes casted to one of the participating parties)	Election database of the Federal Public Service Interior	55.373	12.215
LIT	Local income tax (i.e. levy on personal income taxes collected by the federal government; expressed as a percentage)	Research section of the Flemish government	6.970	1.026
LPT	Local property tax (i.e. levy on property taxes collected by the Flemish Region)	Research section of the Flemish government	1229.400	321.094
Total expenditures	Total expenditures per inhabitant (per year, €)	Research section of the Flemish government	1207.369	410.216
Investments	Investments per inhabitant (per year, €)	Research section of the Flemish government	226.335	143.054
Personnel expenses	Personnel expenses per inhabitant (per year, €)	Research section of the Flemish government	358.855	127.215
Debt	Debt per inhabitant (€)	Research section of the Flemish government	1054.816	558.794
Number of majority parties	Number of parties in the incumbent majority	Election database of the Federal Public Service Interior	1.584	0.629
Income per inhabitant	Income per inhabitant (per year, €)	Statistics Belgium (Federal Public Service Economy)	40444.090	9765.154
Surface area	Surface area of the municipality (hectares)	Research section of the Flemish government	4334.759	2563.561
Inhabitants	Number of inhabitants in the municipality	Research section of the Flemish government	15837.290	11786.640
Ratio of young people to elderly people	Relationship between the number of individuals in the age category of 0–17 years and the number of individuals in the age category of 65 years and older	Research section of the Flemish government	1.212	0.269

Not all of the majority parties appeared with the same name in the elections following their policy terms in 2000, 2006 and 2012. For instance, in certain years, some of these parties formed a cartel that did not exist in other years. Like Vermeir and Heyndels [5], we have therefore chosen not to analyse the election results in such situations. This reduces the theoretical number of observations from 921 (i.e. three results in 307 municipalities) to 580. For 224 municipalities, we have multiple observations (two observations for 114 municipalities and three observations in 110 municipalities). The identification of the FE regression models will be based on these municipalities only. By contrast, the identification of the OLS and 2SLS estimates will also be based on the 81 unique observations for other municipalities.

In our analyses, we focus on six variables relating to municipal budget policy. First, we capture two tax variables: the local income tax (LIT) and the local property tax (LPT). The LIT is a supplementary tax, i.e. it is levied in addition to an existing tax [6]. In this case, the existing tax is the annual personal income tax that the federal government levies on the income of private citizens [7]. More specifically, the municipality adds a supplementary levy to this basic tax in the form of a percentage that it is free to determine. For instance, if the municipal council imposes a tax rate of 7%, the taxpayer must pay €7 to the municipality for every €100 paid in personal income tax. This tax is collected by the federal government, which subsequently transfers the amount due to the taxpayer’s municipality. In 2000, 2006 and 2012, the LIT accounted for about 33% of the total tax revenues of the Flemish municipalities [7].

Every taxpayer who owns property is obligated to pay LPT to the municipality. Each year, the taxpayer receives a notice of assessment for the LPT, which must be paid within the stated period. This amount is claimed by the Flemish Region, and the surcharges are subsequently transferred to the Flemish municipalities [7]. We use the following example to clarify the calculation of the LPT fee [7]. The LPT is calculated based on the indexed cadastral income of a dwelling. Suppose that this indexed cadastral income amounts to €1000 and the municipality has adopted a surcharge–which it is free to determine–of 1300. The first step involves calculating the share for the Flemish Region, which is a fixed fee of 2.5% of the indexed cadastral income. In our example, €25 will go the Flemish Region. This amount is then multiplied by 1% of the LPT surcharge (€25 x 13) to determine the amount to be received by the municipality. In our example, this amounts to €325 per taxpayer [7].

We further include the total expenditures, investment expenditures and personnel expenditures (in the taxpayer’s municipality and in the neighbouring municipalities) as budgetary policy variables. All of these indicators are expressed in euros, per year (in the election year) and per inhabitant. Finally, we examine the effect of the long-term result of the income and expenditure variables (i.e. debt).

All budget variables are rated in December of the election year. This is later than the election month of October, but given that the new elected council is only installed in January of the next year and that budgetary reforms during the last weeks of the incumbent majority is extremely uncommon, this is not expected to be a problem. We observe non-zero variation in the total expenditures, investment expenditures, personnel expenditures and debt among all 224 municipalities with at least two observations. The same is true with respect to LIT and LPT in the neighbouring municipalities. Variation in LIT and LPT, however, is only observed in 123 and 153 municipalities, respectively. The identification of their association with voting outcomes within the FE regression framework will be based on these municipalities only.

Consistent with Vermeir and Heyndels [5], we measure the following instrumental variables for the aforementioned budget variables: surface area, number of inhabitants and the ratio of young people to elderly people (in the municipality and the neighbouring municipalities). The validity of these instruments is conditional on two assumptions: they should be relevant (in a sense that they predict our budget variables) and they should be exogenous (in a sense that they do not have an independent effect on voting outcomes). Indeed, our instruments are expected to correlate with fiscal policy. In particular, recent research has shown that small and large cities compete with a different set of competitors for (mobile) capital, which may yield diverging dynamics in vast and/or densely populated municipalities (versus smaller ones) with respect to fiscal policy [5, 8–9]. Moreover, as a higher proportion of inhabitants below the age of 18 decreases the proportion of tax payers among the population, a positive association between the proportion of young people and tax rates is expected [5, 10]. By contrast, there are no clear indications that the used instruments are endogenous with respect to voting outcomes. As argued by an anonymous reviewer of an earlier version of this article, better incumbents may attract more people who in the end vote for the liked politician while worse incumbents may make some people leave the town and others to vote against. However, homeownership in Flanders is very high so that the tendency to leave one’s municipality is rather low [11]. Therefore, we believe this source of endogeneity may only be relevant at the long term. Nevertheless, both assumptions on which the causal interpretation of our 2SLS results is based are tested empirically. First, with respect to the relevance of our instrumental variables, we conduct F-tests of their joint significance in the first stage of the 2SLS regressions. Second, with respect their exogeneity, we present overidentification tests [12]. Besides these empirical tests, in line with Baert et al. [13], we present a robustness check in which alternative combinations of instruments are used to show that our identification does not hinge on a particular set of instruments.

Given the limited number of instruments, we include no more than three budget variables at a time in the various analyses. Following the primary attention that the literature devotes to the effects of tax outcomes, we always include LIT and LPT as independent variables. These are the only budget variables in the basic model, the estimation results of which are presented in Table 3. In four extended models, the most important coefficients for which are presented in Table 4, we combine LIT and LPT with one of the four other budget variables. This also avoids problems of multicollinearity. In particular, total expenditures are strongly correlated with investments (*Pearson’s r = 0*.*543*), expenditures for personnel (*r = 0*.*703*) and debt (*r = 0*.*408*).

**Table 3:** Basic model: Complete regression results.

Dependent variable	Percentage of votes for majority parties		
Method of estimation	OLS	FE	2SLS
	(1)	(2)	(3)
LIT	-0.305 (0.320)	-0.977 (1.077)	0.725 (1.868)
LIT in neighbouring municipalities	-0.570 (0.800)	-0.416 (2.130)	6.814 (5.476)
LPT	0.001 (0.002)	0.001 (0.004)	-0.014 (0.011)
LPT neighbouring municipalities	0.007** (0.003)	0.004 (0.009)	0.022 (0.013)
Percentage of votes for majority in the previous elections	0.781** (0.049)	0.280** (0.086)	0.721** (0.062)
Number of majority parties	1.385 (0.931)	5.705** (2.000)	2.923* (1.289)
Income per inhabitant/1000	0.093 (0.076)	0.175 (0.233)	0.233 (0.127)
2000 (reference)			
2006	-0.539 (2.891)	-4.133 (4.583)	-4.890 (3.827)
2012	-2.031 (2.853)	-8.339 (6.242)	-9.144* (4.582)
Open VLD in majority in 2012	-2.649 (1.409)	3.309 (2.288)	-3.648* (1.824)
N-VA in majority in 2012	18.764** (1.409)	18.993** (1.877)	17.948**(1.720)
CD&V in majority in 2012	-2.505 (1.695)	6.485* (2.682)	-2.458 (1.963)
Sp.a in majority in 2012	-1.948 (1.631)	4.887 (2.551)	-1.521 (2.026)
GROEN in majority in 2012	-1.677 (2.420)	-0.165 (3.635)	-1.872 (3.123)
Open VLD in majority in 2006	-3.363* (1.804)	4.056 (2.583)	-4.547* (1.980)
N-VA in majority in 2006 (reference)			
CD&V in majority in 2006	4.827** (1.695)	11.279** (2.476)	3.580 (1.855)
Sp.a in majority in 2006	-0.086 (1.661)	0.937 (2.431)	-0.735 (1.993)
GROEN in majority in 2006	-3.078 (2.707)	-1.271 (5.657)	-2.881 (3.565)
Open VLD in majority in 2000	4.859** (1.456)	10.945** (2.582)	2.734 (1.752)
N-VA in majority in 2000	-1.018 (1.938)	-7.443** (2.217)	-2.700 (3.010)
CD&V in majority in 2000	-0.576 (1.456)	4.352 (2.377)	-0.254 (1.923)
Sp.a in majority in 2000	-1.479 (1.458)	-1.700 (2.396)	-1.452 (1.569)
GROEN in majority in 2000	-2.720 (2.591)	-8.227 (11.096)	-0.660 (3.682)
Constant	0.092 (6.930)	21.969 (17.626)	-60.133*(29.592)
Sargan test (p-value)	-	-	0.353
First stage: F-test of instruments’ joint significance (p-value)			
With respect to LIT	-	-	0.019
With respect to LIT in neighbouring municipalities	-	-	0.000
With respect to LPT	-	-	0.000
With respect to LPT neighbouring municipalities	-	-	0.000
Hausman endogeneity test (p-value)	-	-	0.038
N	580	580	580

Notes. The statistics reported are coefficient estimates, with standard errors, clustered at the municipality level, in parentheses.

** (*) indicates significance at the 1%- (5%-) level. OLS stands for ‘ordinary least squares’ (linear regression), FE for ‘fixed effects’ (linear regression with fixed effects at the level of the municipality) and 2SLS for ‘two-stage least squares’ (instrumental variable estimates with six instruments: surface area, number of inhabitants and the ratio of young people to elderly people in the municipality and in the neighbouring municipalities). LIT stands for local income tax and LPT for local property tax.

**Table 4:** Extended models: Main regression results.

Dependent variable	Percentage of votes for majority parties											
Method of estimation	OLS	FE	2SLS	OLS	FE	2SLS	OLS	FE	2SLS	OLS	FE	2SLS
	(1)	(2)	(3)	(4)	(5)	(6)	(6)	(7)	(9)	(10)	(11)	(12)
LIT	-0.637 (0.378)	-1.124 (1.123)	-4.208 (5.187)	-0.282 (0.333)	-0.997 (1.054)	-2.024 (4.670)	-0.495 (0.344)	-1.080 (1.078)	-3.693 (4.581)	-0.477 (0.347)	-0.970 (1.044)	15.699 (46.882)
LIT in neighbouring municipalities	-1.557 (0.920)	-0.560 (2.113)	5.362 (6.803)	-0.853 (0.883)	-0.376 (2.158)	13.801 (14.633)	-0.994 (0.865)	-0.830 (2.184)	3.813 (7.061)	-0.581 (0.814)	-0.749 (2.221)	11.785 (29.977)
LPT	0.001 (0.002)	0.002 (0.004)	0.016 (0.045)	0.001 (0.002)	0.001 (0.004)	-0.002 (0.036)	0.001 (0.002)	0.001 (0.005)	0.025 (0.053)	0.001 (0.002)	0.001 (0.004)	-0.064 (0.241)
LPT in neighbouring municipalities	0.010** (0.003)	0.007 (0.009)	0.001 (0.050)	0.008** (0.003)	0.004 (0.009)	0.009 (0.043)	0.008* (0.003)	0.003 (0.009)	-0.013 (0.055)	0.007* (0.003)	0.004 (0.009)	0.008 (0.127)
Total expenditures	-0.001 (0.001)	-0.001 (0.002)	-0.011 (0.010)									
Total expenditures in of neighbouring municipalities	-0.004 (0.002)	-0.006 (0.004)	-0.004 (0.018)									
Investments				0.002 (0.003)	0.001 (0.004)	-0.034 (0.090)						
Investments in of neighbouring municipalities				-0.010 (0.006)	-0.003 (0.007)	-0.026 (0.209)						
Personnel expenses							-0.005 (0.003)	0.020 (0.018)	-0.031 (0.028)			
Personnel expenses in of neighbouring municipalities							-0.004 (0.004)	-0.002 (0.024)	-0.002 (0.036)			
Debt										-0.001* (0.001)	-0.002 (0.001)	0.043 (0.149)
Debt in of neighbouring municipalities										0.000 (0.001)	0.000 (0.003)	0.057 (0.164)
Additional control variables	Yes	Yes	Yes	Yes	Yes	Yes	Yes	Yes	Yes	Yes	Yes	Yes
N	580	580	580	580	580	580	580	580	580	580	580	580

Notes. The statistics reported are coefficient estimates, with standard errors, clustered at the municipality level, in parentheses.

** (*) indicates significance at the 1%- (5%-) level. OLS stands for ‘ordinary least squares’ (linear regression), FE for ‘fixed effects’ (linear regression with fixed effects at the level of the municipality) and 2SLS for ‘two-stage least squares’ (instrumental variable estimates with six instruments: surface area, number of inhabitants and the ratio of young people to elderly people in the municipality and in the neighbouring municipalities). LIT stands for local income tax and LPT for local property tax. Other variables included: percentage of the incumbent majority during the previous elections, number of majority parties, income per inhabitant, year effect for 2006, year effect for 2012 and the party-year dummies, as listed in Table 3.

Again in line with Vermeir and Heyndels [5], we have included two further characteristics of municipalities in all analyses: number of majority parties (political control variable) and income per capita (economic control variable).

## 3. Results

The results presented in Table 3 and Table 4 are clear. When controlling for municipality fixed effects or when the exogenous variation in the aforementioned instruments is exploited, there are no significant associations between the aforementioned budget variables and the percentage of votes for the parties of the incumbent majority.

Specifically, the linear regression results reveal an association between the percentage of votes for the incumbent majority and two budget components. First, as indicated in Column (1) of Table 3, an increase of 1 (surcharge) unit in the LPT in the neighbouring municipalities improves the percentage of votes for the incumbent majority in the municipality in question by 0.007 percentage points (*p = 0*.*009*). Expressed in terms of standard deviations, an increase of one standard deviation in the LPT in the neighbouring municipalities is associated with a decrease of 0.184 standard deviations in the percentage of votes: 0.007 x 321.094 (standard deviation for the LPT) divided by 12.215 (standard deviation for the percentage of votes). Second, as indicated in Column (1) of Table 4, an increase of €1 per inhabitant in the municipality debt worsens the percentage of votes for the incumbent majority in the municipality in question by 0.001 of a percentage point (*p = 0*.*043*). In addition, a notable second-order result is that, on average, majority parties in broader coalitions did better in subsequent elections in Flanders in 2000, 2006 and 2012 than did majority parties in coalitions consisting of fewer parties.

However, the significance of the aforementioned associations between fiscal policy and voting outcomes is eliminated by either fixed effects regression estimates or instrumental variable regression estimates. Therefore, we must conclude that the associations based on linear regressions are due to the endogeneity of election results and budget policy, as discussed in Section 2.1.

Further analyses indeed empirically indicate that the OLS regression results are biased. First, based on model (3) of Table 3, we performed a Hausman endogeneity test. The assumption of exogeneity of our variables capturing fiscal policy in our basic model is rejected (*p = 0*.*038*). Second, to further investigate the endogeneity of these variables, we run placebo tests, in which we used the percentage of votes for the majority parties in the *previous* elections as an outcome variable. The main results of these analyses are presented in columns (1a) to (1c) of Table 5. These results indicate that at least LPT in neighbouring municipalities is correlated with past values of the outcome, so that we cannot trust the (significant) OLS estimates in Table 3 (and Table 4) and should rely on the fixed effects regression estimates and instrumental variable regression estimates.

**Table 5:** Alternative specifications: Main regression results.

Analysis	1. Placebo test on lagged outcomes			2. Alternative set of instruments			3. Alternative weighting matrix			4. Outcome in differences	
Dependent variable	Percentage of votes for majority parties in the previous elections			Percentage of votes for majority parties			Percentage of votes for majority parties			Percentage of votes for majority parties minus percentage in the previous elections	
Method of estimation	OLS	FE	2SLS	2SLS: exclusion of instruments related to surface area	2SLS: exclusion of instruments related to number of inhabitants	2SLS: exclusion of instruments related to ratio of young people to elderly people	OLS	FE	2SLS	OLS	2SLS
	(1a)	(1b)	(1c)	(2a)	(2b)	(2c)	(3a)	(3b)	(3c)	(4a)	(4b)
LIT	0.261 (0.426)	-0.387 (0.718)	-3.265 (7.399)	-1.618 (6.705)	0.883 (0.9455)	-2.841 (6.686)	-0.167 (0.333)	-0.815 (1.066)	-0.166 (2.181)	-0.128 (1.542)	-16.281 (15.462)
LIT in neighbouring municipalities	-0.222 (0.852)	-1.216 (1.821)	6.362 (11.050)	17.882 (22.211)	-63.021 (200.711)	15.886 (14.991)	-1.562 (0.827)	-2.494 (3.518)	5.040 (5.933)	-5.275 (3.312)	0.391 (26.957)
LPT	-0.000 (0.002)	-0.000 (0.006)	-0.019 (0.063)	-0.021 (0.016)	-0.031 (0.075)	-0.023 (0.020)	0.001 (0.002)	0.001 (0.005)	0.009 (0.010)	0.000 (0.008)	0.157 (0.147)
LPT in neighbouring municipalities	0.007** (0.002)	0.016 (0.013)	0.025 (0.062)	0.024 (0.021)	0.095 (0.214)	-0.008 (0.051)	0.008** (0.002)	0.004 (0.014)	-0.008 (0.014)	0.009 (0.011)	-0.159 (0.157)
Additional control variables	Yes	Yes	Yes	Yes	Yes	Yes	Yes	Yes	Yes	Yes	Yes
N	567	567	567	580	580	580	580	580	580	363	363

Notes. The statistics reported are coefficient estimates, with standard errors, clustered at the municipality level, in parentheses.

** (*) indicates significance at the 1%- (5%-) level. OLS stands for ‘ordinary least squares’ (linear regression), FE for ‘fixed effects’ (linear regression with fixed effects at the level of the municipality) and 2SLS for ‘two-stage least squares’ (instrumental variable estimates with six instruments: surface area, number of inhabitants and the ratio of young people to elderly people in the municipality and in the neighbouring municipalities; except otherwise stated). LIT stands for local income tax and LPT for local property tax. Other variables included: percentage of the incumbent majority during the previous elections (except for column 1), number of majority parties, income per inhabitant, year effect for 2006, year effect for 2012 and the party-year dummies, as listed in Table 3.

By contrast, Table 3 provides empirical support for the two crucial assumptions underlying our 2SLS approach mentioned in Section 2. First, the used instruments are significant predictors of our budget variables–the *p*-value of the related F-tests is always lower than 0.050. Second, the Sargan overidentification test for the 2SLS regression of Table 3 yields a *p*-value of 0.353, supporting the exogeneity of the used instruments with respect to our outcome variable. In addition, in columns (2a) to (2c) of Table 5, we present the main estimation results of three 2SLS models, in which, starting from the specification of model (3) in Table 3, two out of six instruments are dropped. That is, in model (2a) we drop the instruments related to surface area, in model (2b) those related to number of inhabitants and in model (2c) those related to proportion of young inhabitants. Stated otherwise, identification of regressions 2a to 2c of Table 5 is based on four out of the six instruments only. However, our finding of no association between taxes and voting outcomes at the municipality level turns out to be fairly independent of which instruments we use.

Table 5 presents also the results of two further robustness checks of the estimations in Table 3—also the models in Table 4 survive these adaptations (results available on request). First, in models (3a) to (3c), we opt for an alternative weighting scheme in which “neighbouring municipalities” is not operationalised as adjacent municipalities but as municipalities from the same district (with 22 districts of on average 13.95 Flemish municipalities). So, in terms of Eq (1), in this alternative approach, *w*_*ij*_ equals 1 divided by the number of municipalities in the district minus 1 in case *i* and *j* are in the same district and 0 otherwise. Second, as an alternative for our fixed effects approach, we present OLS and 2SLS estimations with the percentage of votes for the majority parties minus their percentage in the previous elections as the dependent variable. Here, we drop the lagged outcome from the right-hand side of the regression model. However, none of these additional analyses yield results that conflict with those discussed above.

Finally, we explore the measured associations at the level of relevant subsamples. That is, we re-estimate the models of Table 3 splitting the data by less and more stable majorities. In columns 1a to 1c (2b to 2c) of Table 6, we present our basic models for the subsample of situations in which the percentage of votes for the incumbent majority in the previous elections was above (below) the overall average of 55.4%. In columns 3a to 3c (4a to 4c), we present the same models for the subsample of observations with a majority comprising more than one (only one) party. For none of these subsamples our regression models taking into account the endogeneity of taxation yield significant associations between tax rates at the municipality level and voting outcomes. The same is true for the additional budget variables included in the models of Table 4 (regression results available on request).

**Table 6:** Subsample analysis: main regression results.

Subsample	1. Percentage of votes for majority in the previous elections ≥ 55.373			2. Percentage of votes for majority in the previous elections < 55.373			3. Number of majority parties ≥ 2			4. Number of majority parties = 1		
Dependent variable	Percentage of votes for majority parties											
Method of estimation	OLS	FE	2SLS	OLS	FE	2SLS	OLS	FE	2SLS	OLS	FE	2SLS
	(1a)	(1b)	(1c)	(2a)	(2b)	(2c)	(3a)	(3b)	(3c)	(4a)	(4b)	(4c)
LIT	-0.560 (0.584)	-1.485 (1.842)	-1.811 (1.545)	-0.009 (0.476)	-0.932 (1.793)	-1.738 (3.751)	-0.718 (0.520)	-0.682 (0.982)	2.437 (2.557)	-0.206 (0.544)	1.028 (1.329)	-0.581 (2.878)
LIT in neighbouring municipalities	-1.130 (1.388)	-2.446 (3.758)	6.026 (12.135)	0.303 (1.194)	4.422 (3.153)	9.761 (10.204)	-1.296 (1.182)	-1.244 (3.721)	-1.344 (5.727)	-1.967 (1.646)	-11.302 (7.999)	-6.051 (8.392)
LPT	0.000 (0.003)	-0.004 (0.008)	0.000 (0.021)	-0.003 (0.002)	0.005 (0.006)	0.005 (0.010)	0.004 (0.003)	0.008 (0.005)	0.020 (0.052)	0.003 (0.003)	-0.004 (0.016)	0.032 (0.027)
LPT in neighbouring municipalities	0.009 (0.005)	-0.002 (0.015)	0.005 (0.012)	0.014** (0.004)	-0.007 (0.016)	0.003 (0.015)	0.002 (0.004)	-0.004 (0.012)	-0.010 (0.026)	0.003 (0.005)	0.037 (0.041)	-0.025 (0.031)
Additional control variables	Yes	Yes	Yes	Yes	Yes	Yes	Yes	Yes	Yes	Yes	Yes	Yes
N	315	315	315	265	265	265	299	299	299	281	281	281

Notes. The statistics reported are coefficient estimates, with standard errors, clustered at the municipality level, in parentheses.

** (*) indicates significance at the 1%- (5%-) level. OLS stands for ‘ordinary least squares’ (linear regression), FE for ‘fixed effects’ (linear regression with fixed effects at the level of the municipality) and 2SLS for ‘two-stage least squares’ (instrumental variable estimates with six instruments: surface area, number of inhabitants and the ratio of young people to elderly people in the municipality and in the neighbouring municipalities). LIT stands for local income tax and LPT for local property tax. Other variables included: percentage of the incumbent majority during the previous elections, number of majority parties (except for columns 4a to 4c), income per inhabitant, year effect for 2006, year effect for 2012 and the party-year dummies, as listed in Table 3.

This result of no significant association between budgetary policy and voting outcomes in Flanders might seem surprising, based on the clear association between certain budget variables and election results in other countries. The results are nevertheless in line with the findings of Van Malderen and Gérard [3] for the neighbouring region of Wallonia. Moreover, our findings correspond to certain anecdotal observations. Whereas budgetary issues are often a point of political dispute and campaigns for elections at higher levels (e.g. regional, federal and European), budgetary policy appears to be much less of an issue in municipal elections in Flanders. Another explanation is that Flemish voters might not have a clear overview of exactly what is included in the income and expenditures of municipalities. For example, as stated previously, the local income tax and local property tax are not collected directly by the municipality, but by the Flemish Region and the federal government, respectively, after which they are transferred. Finally, the various budget variables are clearly interconnected: lower taxes are often accompanied by lower expenditures (and *vice versa*). Flemish voters might be aware of this process of give and take, such that the various budget variables have no independent effects.

## 4. Limitations and directions for future research

The main limitation of this research relates to the causal interpretation of our results. This is only adequate in case the assumptions underlying our fixed effects regression framework and/or our instrumental variable regression framework are met. As mentioned in Section 2, the former approach basically assumes that all unobserved drivers of the election results are constant while the second approach assumes that the included instruments have a direct effect on the budget variables but not on the voting outcomes (after controlling for the other variables included). Although these assumptions are supported by several diagnosis and robustness checks and although our main finding is independent of which particular instruments we use, we cannot fully guarantee causality.

Second, in our regression models, related to our limited number of instrumental variables, we implicitly assume that interaction effects are zero. However, as argued by an anonymous reviewer of an earlier version of this article, voters may take into account the level of expenditures in the municipality when judging tax percentages. Therefore, we are in favour of future studies investigating the potential interaction of certain budgetary variables in affecting election outcomes.

Also the differing results in the literature concerning municipal budget policy and election results call for further research. The conclusion that their association differs by country (and possibly by period) has become clear, and the logical next step should involve explaining this shifting association. Possible moderators of the effect of budget policy on election results can be found at both the macro-level (e.g. powers of municipalities and the level of tax collection) and the micro-level (e.g. the extent to which the population is interested in politics and the economy). We eagerly anticipate future studies that can expose the empirical importance of these moderators.

## 5. Conclusion

In our research, we addressed the relationship between voting behaviour at the municipal level and a broad spectrum–the broadest to date in the literature–of budget elements of municipalities and their neighbouring municipalities: municipal taxes, expenditures (total, for investments and for personnel) and debt. To this end, we analysed 580 election results in Flanders. Our results indicate that, in the voting booth, Flemish voters do not give any substantial consideration to the financial policies of their municipalities. These results can be given a causal interpretation conditional on the assumptions underlying our fixed effects regression framework (i.e. unmeasured determinants of both tax policy and voting outcomes are time-constant) and/or our instrumental variable regression framework (i.e. our instruments, or at least a subset of them, are relevant in explaining tax policy and exogenous with respect to voting outcomes).

## Supporting information

S1 Dataset (XLS)
